# COVID-19–ASSOCIATED HOSPITALIZATIONS AMONG ADULTS AGES ≥75 YEARS—COVID-NET, OCTOBER 2022–FEBRUARY 2023

**DOI:** 10.1093/geroni/igad104.1487

**Published:** 2023-12-21

## Abstract

Older adults are at increased risk for COVID-19-associated hospitalizations and other severe outcomes. Recent hospitalizations among adults ages ≥75 years were described using data from CDC’s COVID-19-Associated Hospitalization Surveillance Network (COVID-NET), a population-based surveillance network of >300 acute-care hospitals across 14 U.S. states. Percentages were statistically weighted as necessary to represent the underlying population of the surveillance catchment area, unless noted. From October–December 2022, population-based hospitalization rates among adults ages ≥75 years were the highest of all age groups and ≥3 times as high as rates among the next oldest age group, adults ages 65–74 years. During that same period, adults ages ≥75 years comprised 42% (unweighted) of the 13,721 laboratory-confirmed adult hospitalizations identified in COVID-NET. Among a stratified random sample of adults ages ≥75 years (N=391), the most common underlying medical conditions present upon admission included chronic lung disease (72%), cardiovascular disease (65%), neurologic disorders including dementia (45%), and diabetes (36%). Among adults ages ≥75 years, 9% had received the updated booster and 74% were vaccinated but had not received an updated booster dose. Adults ages ≥75 years comprise an increasingly disproportionate share of COVID-19-associated hospitalizations with the majority of recent hospitalizations occurring among those who have not received an updated COVID-19 booster dose. Underlying medical conditions can increase the risk for hospitalization and other severe outcomes and are common in this group. Receipt of updated COVID-19 bivalent booster doses and early antiviral treatment to prevent hospitalization are essential for this age group.

